# Induction of antitoxic antibody and preventive effect against porcine edema disease by the pentameric Stx2eB subunit vaccine

**DOI:** 10.1186/s13567-023-01161-1

**Published:** 2023-03-27

**Authors:** Masaya Yoshimura, Youko Honda, Emi Yonemitsu, Ryota Takahashi, Kiyotaka Suenaga, Takashi Waki

**Affiliations:** 1 Meiji Animal Health Co., Ltd., 1-6-1 Okubo, Kita-Ku, Kumamoto-Shi, Kumamoto, 860-0083 Japan; 2 KM Biologics Co., Ltd., 1-6-1 Okubo, Kita-Ku, Kumamoto-Shi, Kumamoto, 860-8568 Japan

**Keywords:** Porcine edema disease, Shiga toxin 2e, vaccine, Stx2e neutralizing antibody, Stx2e B subunit, cartilage oligomeric matrix protein

## Abstract

Porcine edema disease (ED) is an enterotoxaemia that frequently occurs in 4–12 week-old piglets and results in high mortality. ED is caused by Shiga toxin 2e (Stx2e), produced by host-adapted Shiga toxin-producing *Escherichia coli* (STEC) strains. We constructed a recombinant protein in which the B subunit of Stx2e (Stx2eB) was linked to Cartilage Oligomeric Matrix Protein (COMP)’s pentameric domain to enhance antigenicity to induce neutralizing antibodies against Stx2e. We evaluated the efficacy of this antigen as a vaccine on the farm where ED had occurred. The suckling piglets were divided into two groups. The pigs in the vaccinated group were intramuscularly immunized with the vaccine containing 30 µg/head of Stx2eB-COMP at 1 and 4 weeks of age. The control pigs were injected with saline instead of the vaccine. The neutralizing antibody titer to Stx2e, mortality, clinical score, and body weight was evaluated up to 11 weeks after the first vaccination. In the vaccinated group, the Stx2e neutralizing antibody was detected 3 weeks after the first vaccination, its titer increased during the following weeks. The antibody was not detected in the control group during the test period. The STEC gene was detected in both groups during the test period, but a typical ED was observed only in control pigs; the mortality and clinical score were significantly lower in the vaccinated group than in the control group. These data indicate that the pentameric B subunit vaccine is effective for preventing ED and offers a promising tool for pig health control.

## Introduction

ED is an enterotoxaemia caused by Stx2e that frequently occurs in 4–12 week-old piglets. It is clinically characterized by swelling of the eyelids, paralysis, unusual voice, neurologic signs such as ataxia, lateral recumbency, and high mortality (50–90%) [1]. Therefore, ED causes great economic losses to pig farming.

ED effected pigs (or piglets) are treated with antibiotics, but the efficacy is poor because Stx2e has already been absorbed in the blood stream when clinical signs become apparent. Certain antibiotics such as quinolones, trimethoprim, and furazolidone could induce SOS response, by increasing the transcription of Stx2 genes [2]. In addition, as multidrug-resistant bacteria have been reported, antibiotics need to be selected appropriately [1]. As an alternative method, high amounts of zinc oxide may be added to feed [3], but there are concerns about the increase of drug-resistant bacteria and environmental pollution by zinc. Therefore, the exploitation of other control tools for ED, such as vaccines, is desired.

Stx2e is categorized in the Stx2 group of Shiga toxins. It has been reported as a developmental factor and protective antigen of ED vaccines containing Stx2e toxoid or genetically detoxified Stx2e by mutating the active toxin sites [4–8]. Although the vaccines have been confirmed to be effective against ED, they have shortcomings, such as a risk of side reactions due to the remaining toxin activity in the toxoid. Also, the yields of both vaccines were too low for commercial use [7–10].

**Figure 1:**
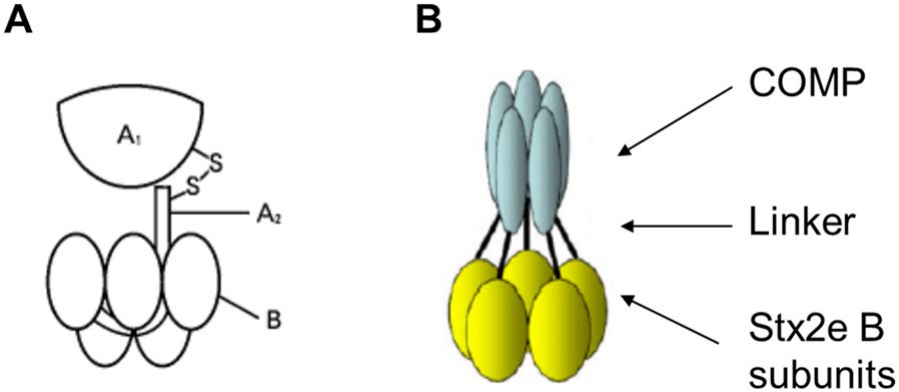
**Structure of Shiga toxin and Stx2eB-COMP.** **A** All Shiga toxins have the AB5 toxin structure. A subunit is cleaved into A1 and A2 (held together by disulfide bonds). The five small B subunits surround the A2 region. **B** A structural diagram of Stx2eB-COMP. It was produced by fusion with COMP, also a pentamer, to stabilize the pentamer structure of the B subunit.

ED is caused by STEC colonizing the small intestine [1]. Shiga toxin is an AB5 toxin protein (Figure 1A) and is transferred from the intestine into the bloodstream. It binds to a receptor on the surface of vascular endothelial cells via the B subunits. Then, the A subunit is internalized by receptor-mediated endocytosis. The A subunit having rRNA N-glycosidase activity induces cell death by inhibiting ribosomal protein synthesis [11].

We hypothesized that a vaccine could be developed to prevent the onset of ED by blocking the binding of Stx2e to the cell surface receptor. Therefore, we used the B subunit having cell receptor binding ability as a vaccine antigen. Furthermore, as described above, since the Shiga toxin has the structure of AB5, we considered it important to maintain the B subunits as an immunizing antigen. Therefore, we created a recombinant protein (Stx2eB-COMP, Figure 1B) by fusing a Stx2e B subunit and a coiled-coil forming unit derived from COMP. COMP is a pentameric structure of oligomeric glycoprotein synthesized by chondrocytes [12]. In this study, to induce neutralizing antibodies against Stx2e, we developed a vaccine containing Stx2eB-COMP antigen as an oil-in-water emulsion; and evaluated the efficacy of this vaccine on a farm where ED had occurred.

## Materials and methods

### Construction of Stx2eB-COMP expressing vector and preparation of Stx2eB-COMP expressing* E. coli*

The nucleotide sequence for Stx2eB was artificially synthesized based on a Stx2eB gene (GenBank: NC_003356.1) which optimized the codons for expression in *E. coli* and yeast. First, the synthetic nucleotide was digested with *Nde*I and *Xho*I, and ligated into a plasmid pET-22b (Merck KGaA) to generate a plasmid pStx2eB. Next, the nucleotide sequence for matured Stx2eB containing no secretory signal sequence was amplified by PCR using a plasmid pStx2eB and primers (sense, 5′-CATGCCATGGATTGCGCGAAAGGCAAAATTG-3′ and antisense, 5′-CCGCTCGAGATTGAATTTCACCTGCGCAAAAC-3′). Finally, the amplified fragment was digested with *Nco*I and *Xho*I, and ligated into a plasmid pET-21d (Merck KGaA) to generate a pSTXB plasmid.

The nucleotide sequence for a fusion protein of (GP)_2_GH_6_(G_4_S)_3_ linker and COMP was amplified by PCR using a plasmid pB-spacer which includes nucleotide sequence for (G_4_S)_3_-COMP [13] and primers (sense, 5′-GGGCTCGAGGGTCCAGGACCTGGACATC-3′ and antisense, 5′-GGGCTCGAGTCAGCCCGGGGTACGGGCC-3′). The amplified fragment and a pSTXB plasmid were treated with *Xho*I and joined to generate a plasmid pSTXC. The pSTXC plasmid is a vector for expressing a fusion protein of Stx2eB, the (GP)_2_GH_6_(G_4_S)_3_ linker, and COMP. *E. coli* BL21(DE3) strain transfected with pSTXC, generates an *E. coli* strain STXC which expresses Stx2eB-COMP protein.

### Cultivation of *E. coli* strain STXC and purification of expressed protein

*E. coli* strain STXC was cultured in YE broth containing 50 µg/mL of ampicillin for 6 h at 37 °C. After adding isopropyl-β-d-thiogalactoside to express Stx2eB-COMP protein, it was further cultured for 40 h. After centrifugation, the harvested cells were washed with cell wash buffer (50 mM Tris–HCl (pH 8.0) containing 1 mM EDTA and 100 mM NaCl). The cells were subjected to high-pressure crushing, and the recovered centrifugal sediment was washed with protein-wash buffer (50 mM Tris–HCl (pH 8.0) containing 100 mM EDTA, 2% Triton X-100, and 2 M Urea) and distilled water. The obtained insoluble protein was solubilized with an unfolding buffer (20 mM Na_2_HPO_4_ (pH 8.2) containing 6 M guanidine hydrochloride (GuHCl) for 24 h at 37 °C, and then refolded with refolding buffer (20 mM Na_2_HPO_4_ (pH 8.2) containing 1 M arginine hydrochloride and 2 mM l-cystine) for 24 h at 25 °C. The protein solution was concentrated and the medium was replaced with PBS (pH 8.0) by ultrafiltration and then filtered using a 0.22 μm filter. The obtained Stx2eB-COMP protein was used as a vaccine antigen. The Stx2eB-COMP protein concentration was determined by densitometry (Quantity one ver.4.6, BIO-RAD, CA, US) after gel electrophoresis.

### Vaccine

An oil-in-water type emulsion vaccine containing 30 µg of the Stx2eB-COMP protein in 1 mL per dose was prepared according to the following procedure. Liquid paraffin (Kaneda Co., Ltd, Tokyo, Japan) adjuvant and emulsifiers of Polysorbate 80 (NOF Corporatin, Tokyo, Japan) and sorbitan monooleate (NOF Corporatin, Tokyo, Japan) were mixed with PBS and emulsified using a homogenizer. One volume of Stx2eB-COMP antigen was mixed with two volumes of emulsified adjuvant and used as a trial vaccine.

### Farm trial and its ethical statement

The test was conducted on a farm where ED had occurred. The 159 healthy crossbred suckling piglets (Landrace, Large-White, Duroc) at 1 week of age were divided into two groups without weight and gender bias. The vaccinated group (male: 40, female: 39) was injected with 1 mL of vaccine into the neck muscle at a 3-week interval, and the control group (male: 40, female: 40) was injected with 1 mL of saline as the vehicle. After weaning at 4 weeks of age, efficacy was evaluated until 11 weeks after the first vaccination (12 weeks old). Piglets were bred during the test period in a facility with constant feeding and water supply. This clinical trial was conducted in compliance with the “Act on Welfare and Management of Animals” [14] and following the KYODOKEN Institute guidelines for animal studies.

### Detection of STEC

STEC was identified by PCR targeting the stx2e gene. Small intestinal contents and mesenteric lymph nodes collected at the time of death, and fecal swabs were used as test samples. The fecal swabs were collected from piglets that indicated fecal abnormalities during the test period.

### Purification of Stx2e

STEC was cultured in YE broth overnight at 37 °C. The STEC culture suspension was centrifuged, and the precipitate was suspended in 20 mM phosphate buffer (pH7.0). The harvested cells were sonicated and centrifuged, the supernatant was purified by affinity chromatography (Cellfine™ Sulfate, JNC Corporation, Tokyo, Japan), and then dialyzed with PBS. Purified Stx2e was filtered with a 0.22 µm filter and stored frozen.

### Stx2e neutralizing antibody assay

Sera were treated for 30 min at 56 °C before use, and then serially diluted twofold with supplemented Eagle minimal essential medium (E-MEM, Nissui Pharmaceutical Co., Ltd, Tokyo, Japan) containing 5% fetal bovine serum, 3.0 mg/mL trypticase soy broth (Becton, Dickinson and Company, Tokyo, Japan), 1.1 mg/mL sodium bicarbonate (FUJIFILM Wako Pure Chemical Corporation. Osaka, Japan), 2 mM l-glutamine (FUJIFILM Wako Pure Chemical Corporation. Osaka, Japan), 100 U/mL penicillin (Meiji Seika Pharma Co., Ltd, Tokyo, Japan), and 100 µg/mL streptomycin (Meiji Seika Pharma Co., Ltd, Tokyo, Japan). Then, 10 CD_50_ (median cytotoxic dose) of purified Stx2e were added to each serum sample and the toxin-antiserum mixtures were incubated at 37 °C for 1 h. Toxin-antiserum mixture (50 µL) was added to 96-well plates containing 100 µL of supplemented E-MEM, followed by 50 µL (4 × 10^5^ cells/mL) of Vero cells (ATCC CCL-81) added to each well, and the plates were incubated at 37 °C in 5% CO_2_ for 5 days. The cell viability was assessed using the Cell Counting Kit-8 (CCK-8, DOJINDO LABORATORIES, Kumamoto, Japan). CCK-8 solution (50 µL) diluted 11 times with supplemented E-MEM was added into each well. The cultures were incubated at 37 °C in 5% CO_2_ for 2 h. The absorbance of each well was measured at 450 nm using a microplate reader (SUNRISE RAINBOW THERMO, Tecan Japan Co., Ltd., Kanagawa, Japan). When the value obtained by dividing the absorbance value of the sera by that of the control well (no toxin addition) was more than or equal to 0.5, the neutralizing effect of Stx2e was deemed positive. The neutralizing antibody titer was the reciprocal of the highest dilution of serum which had a Stx2e neutralizing effect.

### Assessment of vaccine efficacy

Stx2e neutralizing antibody titer, mortality, clinical score, and body weight of the groups were compared to evaluate the vaccine efficacy. Sera were collected from 30 heads of the vaccinated group and 10 heads of the control group at 0, 3, 7, and 11 weeks after the first vaccination. The Stx2e neutralizing antibodies in serum were detected by the Stx2e neutralizing antibody assay. Clinical signs were scored between 0 and 11 weeks after the first vaccination using the criteria shown in Table 1 [15]. The body weights of all animals were measured at weeks 0, 3, 7, and 11 after the first vaccination.

**Table 1:** **Criteria of clinical scores**

Clinical signs	Clinical scores			
	0	1	2	3
Vitality	Good	Loose	Bad	–
Appetite	Good	Slightly poor	Poor	None
Respiration	Normal	Slightly quick	Quick	–
Palpebral edema	Normal	Mild	Moderate	Severe
Feces	Normal	Loose stool	Moderate diarrhea	Severe diarrhea
Neurologic impairment	Not impairment	Slightly	Moderate	Severe
Piloerection	None	Slightly	Moderate	Severe

### Statistical analysis

Mortality was tested using the Fisher exact test. The body weight was subjected to an F test followed by the Student *t*-test or Aspin-Welch *t*-test. The clinical scores were evaluated using the Mann–Whitney U test. The significance level was ˂ 5% in all cases.

## Results

### Mortality

Seven out of eighty piglets showed clinical signs such as palpebral edema, intestinal edema/hyperemia, and mesenteric lymphadenopathy and died between days 38 and 67 in the control group (Table 2). The symptoms observed in these piglets were characteristic of edema disease. In the vaccinated group, seventy-nine piglets showed significantly lower clinical signs (scores) compared to the control group, and no piglets died. The mortality rates (%) were 0.0 (0/79) in the vaccinated group and 8.8 (7/80) in the control group, showing a significant difference in mortality between the groups.

**Table 2:** **Detection of STEC from dead piglets in the control group**

Animal ID^*1^	Date of death	Small intestine	Lymph nodes
43	38	−	+
51	38	−	+
122	50	−	N.T.^*2^
148	51	−	N.T
156	51	−	N.T
135	67	−	+
158	67	−	+

^*1^: Eight out of a total of 80 piglets died during the study period.

^*2^: N.T. means “not tested”.

### Clinical score

**Figure 2:**
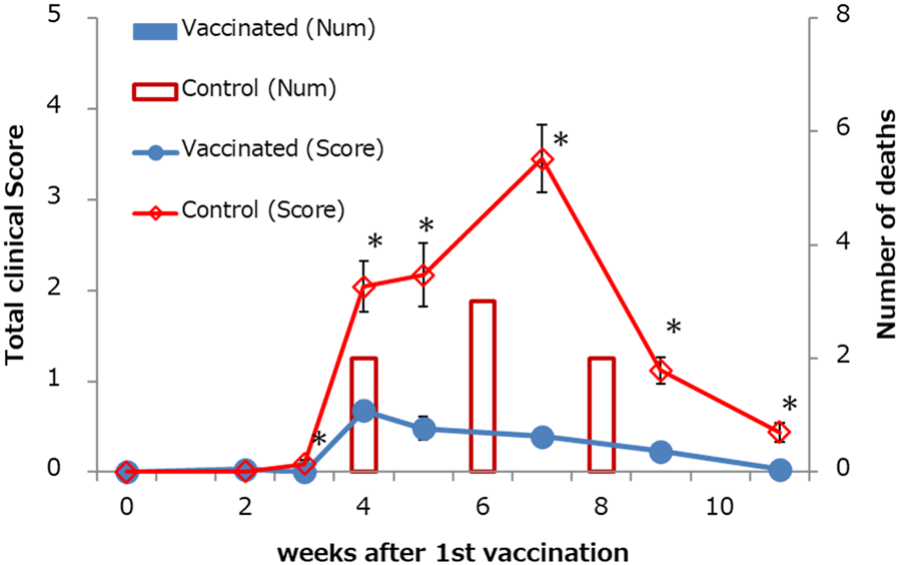
**No vaccinated piglets died and showed low clinical scores.** The total clinical score (mean ± SEM) at 0, 2, 3, 4, 5, 7, 9, 11 weeks after 1^st^ vaccination and the number of deaths during the test period are shown. *: There are significant differences (*P* < 0.05) in the clinical scores of the two groups.

As described in the sub-section “Mortality”, only control pigs, but no vaccinated pigs died. The clinical score increased in both groups, but the score of the vaccinated group remained lower than that of the control group (Figure 2). There were significant differences in clinical scores between the groups from weeks 3 to 11 after the first vaccination. In the vaccinated group, the clinical score of feces were significantly lower during 3 to 4 weeks after the first vaccination, and from week 5 onwards, other clinical scores were significantly lower than those of the control group.

### Body weight gain

Body weight was not significantly different between the two groups at 3 weeks after the first vaccination. However, it was significantly higher in the vaccinated group than in the control group at 7 (18.8 kg vs. 17.4 kg) and 11 weeks (35.7 kg vs. 31.8 kg) after the first vaccination (Table 3).

**Table 3:** **Trends in body weights (kg, mean ± SEM) during the test period**

Group	Weeks after 1^st^ vaccination			
	0	3	7	11
Vaccinated	3.0 ± 0.1	7.0 ± 0.1	18.8 ± 0.3^a^	35.7 ± 0.5^a^
Control	2.9 ± 0.1	7.0 ± 0.1	17.4 ± 0.5^b^	31.8 ± 0.6^b^

Significant differences (*P* < 0.05) were represented by different superscript letters at each time point.

### Detection of STEC

STEC was detected in fecal swabs collected from the control group between 23–41 days after the first vaccination and from the mesenteric lymph nodes of dead piglets during the test period (Tables 2 and 4). In the vaccinated group, STEC was only detected in fecal swabs collected on day 34 after the first vaccination. The degree of abnormality in the fecal samples varied from clinical score 1 (loose stool) to 3 (severe diarrhea).

**Table 4:** **Detection of STEC from abnormal fecal swabs**

Group	No. of samples	No. of positive	Days elapsed since the 1^st^ vaccination
Vaccinated	12	1	34
Control	35	6	23–41

### Stx2e neutralizing antibody

The Stx2e neutralizing antibody titer increased from the week 3 after the first vaccination and showed a peak value of 88.4 at week 7 after the first vaccination. On the contrary, the titer of the control group was negative during the test period despite the onset of symptoms and subsequent death.

## Discussion

We developed a trial vaccine consisting of a purified Stx2eB-COMP protein in an oil-in-water emulsion and evaluated its efficacy in a trial on a farm where ED had occurred. The neutralizing antibody titer increased significantly in the vaccinated group. The group showed a lower clinical score and higher body weight than the control group. STEC was detected from fecal swabs of both groups, meaning that STEC was spread throughout the farm during the test period. However, the mortality in the vaccinated group was 0%, while that in the control group was 8.8%. These data indicate that this vaccine candidate can prevent the onset of ED. Moreover, no serious adverse reactions were observed after administering this trial vaccine.

This study focused on the Stx2e B subunit as a vaccine antigen to block toxin binding. We confirmed the vaccine efficacy due to the antibody to the B subunit induced by immunization with the Stx2eB-COMP protein that inhibited the binding of Stx2e to the receptor on the surface of vascular endothelial cells. Meanwhile, it has been reported that the B subunit alone induces little of the Stx2e neutralizing antibodies compared to the holotoxin [16]. In addition, the sera obtained by immunizing Stx2 B subunit monomers could not recognize Stx2 [17]. In our previous study, we compared neutralizing antibody induction by Stx2eB alone and Stx2eB-COMP using 7-week-old BALB/c mice. The result confirmed that the Stx2eB-COMP antigen induces neutralizing antibodies against Stx2e in mice, while no increase in neutralizing antibodies was observed in the Stx2eB alone injection group [18]. These reports and data suggest that the multimeric structure of the B subunit is important for the maintenance of neutralizing epitopes. Therefore, we considered that the pentameric structure of the B subunit was important for inducing Stx2e neutralizing antibodies efficiently; therefore, we produced the Stx2eB-COMP.

Clinical scores except those of feces were significantly inhibited at 7 weeks post-vaccination. The difference in clinical scores between vaccinated and control groups was the highest at this time point. These clinical symptoms, such as vitality, appetite, respiration, palpebral edema, neurologic impairment, and piloerection are considered to be due to Stx2e-induced toxemia. This inhibitory effect is presumed to be due to the neutralization of the toxin by the induced antibodies, suggesting that the induction of neutralizing antibodies against Stx2e is important for ED vaccines.

Although STEC were isolated from the control group, the Stx2e neutralizing antibody remained negative in this group during the test period. Gannon et al. showed previously that no Stx2e neutralizing antibody was detected in swine herds suffering from ED [19], confirmed in this study since no neutralizing antibody was detected even after STEC infection. Additionally, although the animal species are different, when a STEC oral infection experiment was conducted in cattle, the antibody against Stx1 increased rapidly in adult cattle (steered cattle), but gradually in calves. Moreover, no antibody response to Stx2 was developed [20]. It is inferred from these data that Stx2 has an extremely weak antibody-inducing ability in living organisms. Therefore, we believe that our vaccine is promising because it can induce neutralizing antibodies that are normally difficult to produce in STEC infection in piglets.

In conclusion, our trial vaccine induced Stx2e neutralizing antibodies efficiently using only the B subunits of Stx2e and effectively prevented ED. The result indicates that the A subunit is not essential for ED prevention. Since the antibodies against Stx2e were not able to inhibit STEC from developing, the clinical signs shown in some of vaccinated piglet might be caused by exceeded levels of un-neutralised Stx2e. These results indicate that this vaccine can be a new tool for prevention of ED exacerbation by Stx2e.

## Data Availability

The datasets of this clinical study are available from the corresponding author upon reasonable request.
